# Multi-scale object detection in UAV images based on adaptive feature fusion

**DOI:** 10.1371/journal.pone.0300120

**Published:** 2024-03-27

**Authors:** Siqi Tan, Zhijian Duan, Longzhong Pu

**Affiliations:** School of Traffic and Transportation, Chongqing Jiaotong University, Chongqing, China; Balochistan University of Engineering and Technology Khuzdar, PAKISTAN

## Abstract

With the widespread use of UAVs, UAV aerial image target detection technology can be used for practical applications in the military, traffic planning, personnel search and rescue and other fields. In this paper, we propose a multi-scale UAV aerial image detection method based on adaptive feature fusion for solving the problem of detecting small target objects in UAV aerial images. This method automatically adjusts the convolution kernel receptive field and reduces the redundant background of the image by adding an adaptive feature extraction module (AFEM) to the backbone network. This enables it to obtain more accurately and effectively small target feature information. In addition, we design an adaptive feature weighted fusion network (SBiFPN) to effectively enhance the representation of shallow feature information of small targets. Finally, we add an additional small target detection scale to the original network to expand the receptive field of the network and strengthen the detection of small target objects. The training and testing are carried out on the VisDrone public dataset. The experimental results show that the proposed method can achieve 38.5% mAP, which is 2.0% higher than the baseline network YOLOv5s, and can still detect the UAV aerial image well in complex scenes.

## 1. Introduction

In recent years, the rapid popularization and development of UAVs have led to their wide applications in many fields. UAV aerial images can be used to detect and track targets, and can provide important information for traffic planning, military operations, and personnel rescue. Therefore, it is of great practical significance to study the target detection method of UAV aerial images. However, the detection of targets in UAV aerial image presents several challenges, the most prominent of which is the detection of small target objects. Due to the change of the height and angle of the UAV in the process of aerial photography, the scale of the target object in the obtained image often changes to a certain extent. In addition, the pixels of the original small target objects are relatively small in the image, and the image background also produces different degrees of interference in the process of detection. These factors make it particularly difficult to obtain features of small targets accurately and effectively.

At present, the detection methods proposed for solving the inability of UAV aerial images to detect small targets are mainly divided into two types. One is the traditional detection method. This method entails manually extracting the features of each detection target in the aerial image, so as to ensure the accuracy and effectiveness of the obtained feature information of the detected target. The process is cumbersome and the robustness of the model is poor. The other is a detection method based on deep learning, in which neural network is used instead of artificial method to extract features of each detection target in the image. It has high flexibility, strong robustness and less artificial quantity. In recent years, due to the rapid development of artificial intelligence technology and neural network, the target detection network of UAV aerial image based on deep learning has greatly exceeded the traditional detection method in terms of detection accuracy and detection speed. However, there are still some problems in the detection of small targets, such as low detection accuracy, easy missed detection and false detection. Through analysis and research, we propose a multi-scale UAV aerial image target detection method UAV-YOLO based on adaptive feature fusion.

The rest of this paper is organized as follows: The second section mainly reviews the works related to the current UAV aerial image target detection method. The third section proposes a multi-scale UAV aerial image target detection method based on adaptive feature fusion. The fourth section introduces the experimental process of the proposed method on the VisDrone public dataset in detail, and the experimental results are discussed and analyzed. The fifth section gives the final conclusion of this paper and the future research direction.

## 2. Related works

### 2.1. Two-stage UAV aerial image target detection method based on proposed region

Currently, the deep learning network methods used to detect UAV aerial images are mainly divided into two categories. One is a two-stage method based on the proposed region, and the other is a single-stage method based on regression. The two-stage detection method based on the proposed region first uses the UAV aerial image to generate the corresponding proposed region, then extracts the features of the targets contained in the proposed region, and finally classifies and regresses the targets according to the extracted feature information. The representative networks based on this method type are Fast R-CNN [1], Faster R-CNN [2], Mask R-CNN [3], etc. At present, many scholars use the two-stage method to study the target detection of UAV aerial images. MITTA et al. [4] address the difficulty of extracting features of low-altitude targets in aerial images by adding a Dilated ResNet Module (DRM) based on the R-CNN network to obtain the context semantic information of low-altitude small targets. This method can better detect small targets, but it cannot ideally detect multi-type targets. R. Jin et al. [5] merged the regions where small targets are located in the image and trained these regions to improve the detection effect of the network on small targets. This method has good detection effect on targets of various scales, but the detection process is cumbersome and complex, and it is difficult to deploy on the mobile terminal. Junfeng Wan et al. [6] improved the Feature Pyramid Network (FPN) and region of interest (ROI) to enhance the network ’s ability to extract each target feature in the image. Benefitting from the various enhancement strategies, the detection effect of the network on various targets is slightly improved, but its detection speed and real-time performance are poor. Sungeun Hong et al. [7] proposed a hard chip mining method to solve the problem of unbalanced proportion of target categories in aerial images. It improves the accuracy of the network by generating additional hard examples for training. The method achieves good detection results, but the detection speed of the model is low. Haoran Wang et al. [8] added a newly designed Receptive Field Expansion Block (RFEB) and a Spatial-Refinement Module (SRM) to DetNet [9] to enhance the high-level semantic features of each detected target in the aerial images as well as to repair the details of the acquired multi-scale features. This method can improve the detection accuracy of the network, but it also leads to a dramatic increase in the number of model parameters and a significant decrease in the detection speed. Ziming Liu et al. [10] proposed HRDNet for detecting high-altitude small target objects. In this network, high-resolution and low-resolution images are processed using the designed Multi-Depth Image Pyramid Network (MD-IPN) and Multi-Scale Feature Pyramid Network (MS-FPN) to preserve more target position information. This method can effectively detect small target objects, but the detection speed of the network is slow and cannot meet the requirements of real-time detection. Rebbapragada V C Sairam et al. [11] designed ARchitectUre-agnostic BAlanced Loss (ARUBA) to improve ReDet [12] to solve the problem of imbalance in the size of the detected targets in each category in UAV aerial images. The method can improve the network’s detection of each target, but the enhancement of detection of small target objects is small.

### 2.2. A single-stage UAV aerial image target detection method based on regression

The single-stage detection method based on regression considers the target detection of UAV aerial images as a regression problem. It does not need to generate the corresponding proposed region, but directly performs feature extraction and classification regression on the entire image, thereby achieving end-to-end target detection. The representative networks of these methods are SSD [13], YOLO [14], RetinaNet [15], etc. Many studies that are done on the target detection of UAV aerial images are based on this method. PEI W et al. [16] addressed the problem of UAV aerial image being prone to miss targets by using the residual network with stronger learning ability to improve the SSD detection network, and efficiently fused the feature information in a hierarchical way. Although this method reduces the rate of missing targets, the deep network structure leads to poor real-time detection. S. Feng et al. [17] improved yolov3 by using hybridizing attention mechanism and Spatial Pyramid Pooling (SPP) network, which enhanced the representation of small target feature information through attention mechanism. This method can effectively improve the detection effect of the network for small target objects, but the overall detection accuracy and detection speed of the network need to be improved. In addition, ALI et al. [18] addressed the problem of detecting small targets in UAV aerial images by modifying the YOLOv4 detection network. In this network, more small target feature information is obtained by splicing the up-sampling features with the original features. The detection speed of this method is fast, but the detection accuracy needs to be improved. Kyoungtaek Choi et al. [19] compressed and pruned the channels and residual blocks of the yolov4 network model and deployed them into an embedded device to achieve real-time detection of small target objects. Although this method solves the deployment problem of the network model to a certain extent, the accuracy and speed of detection still need to be improved. Bi-Yuan Liu et al. [20] proposed ZoomInNet, which improves the detection accuracy of each target type while reducing the number of model parameters by refining the feature information at different scales and then compressing it. However, the feature information that can be extracted by this method is limited, which does not improve the detection performance of the model significantly.

### 2.3. Our work

Although there are many detection methods for UAV aerial images, most of them are difficult to achieve a good balance between detection accuracy and real-time detectability. Higher detection accuracy often results in larger models and poor real-time detectability, but maintaining smaller models often results in lower detection accuracy.

To accurately and efficiently detect UAV aerial images, this paper proposes a multi-scale UAV aerial image target detection method based on adaptive feature fusion by combining the characteristics of aerial images with more small targets. The method combines the attention module and the convolutional kernel with the adjustable sensory field, which improves the efficiency of small target feature extraction while reducing the computation amount of the image background. In addition, an adaptive feature fusion method for small target shallow feature information is designed to reduce the loss of small target shallow feature information. Finally, this paper also adds additional small target detection scales to improve further the network’s ability to sense small targets. The above structural design enables the network model size to achieve better detection accuracy while maintaining a small level.

## 3. Proposed multi-scale object detection network based on adaptive feature fusion

### 3.1. Proposed network architecture

**Fig 1:**
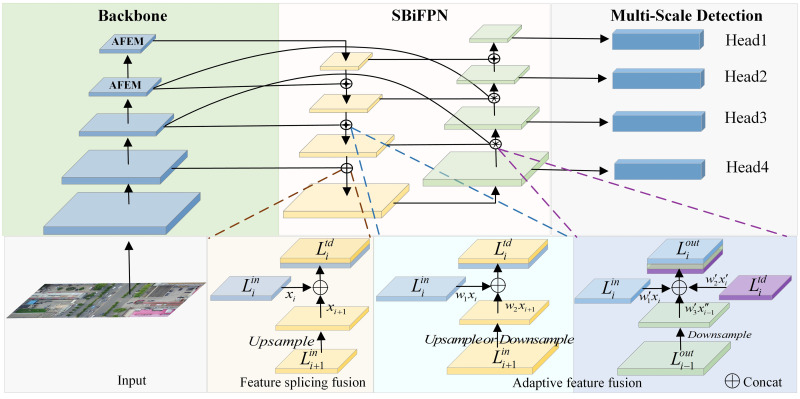
The proposed UAV-YOLO network architecture.

On the basis of fully ensuring the real-time detection performance of the network, a multi-scale UAV aerial image target detection method based on adaptive feature fusion (UAV-YOLO) is proposed to solve the problem of not detecting many small target objects in UAV aerial images. The proposed detection method is based on YOLOv5s, and the overall architecture is shown in Fig 1. It mainly includes the following three parts:

- **Feature extraction backbone network**. An adaptive feature extraction module (AFEM) is added to the network. The AFEM module first adaptively adjusts the receptive field of the convolution kernel through deformable convolution, so that the network can obtain more small target feature information. Secondly, the coordinate attention is used to reduce the interference of redundant background in the image, so that the network can accurately and effectively extract the features of small target objects and reduce the network computing overhead.
- **Adaptive feature fusion network**. The network reduces the loss of shallow feature information in the convolution process by adaptively weighting and fusing the feature information obtained by the feature extraction network, thereby enhancing the expression of small target features in UAV aerial images.
- **Multi-scale detection network**. In the network part, the receptive field of the network for small targets is improved by adding additional small target detection scales. This enables the network to predict the targets in the UAV aerial images from four scales, thereby improving the detection effect of the network on the UAV aerial images.

### 3.2. Adaptive feature extraction module

To extract the feature information of the target from the UAV aerial image, the image needs to be fed into the convolutional neural networks. Through the repeated convolution of the convolutional neural network, the shallow and deep features of the detected target are obtained. Due to the change of shooting angle and height during the flight of the drone, the size of small targets in the total pixels of the image is small and the targets in the image usually undergo different degrees of deformation. Extracting features from small target objects can be difficult due to issues like deformation of objects in the image and the redundant background. This would make the network not to accurately and effectively obtain features of small targets, and the computational overhead also increases significantly.

**Fig 2:**
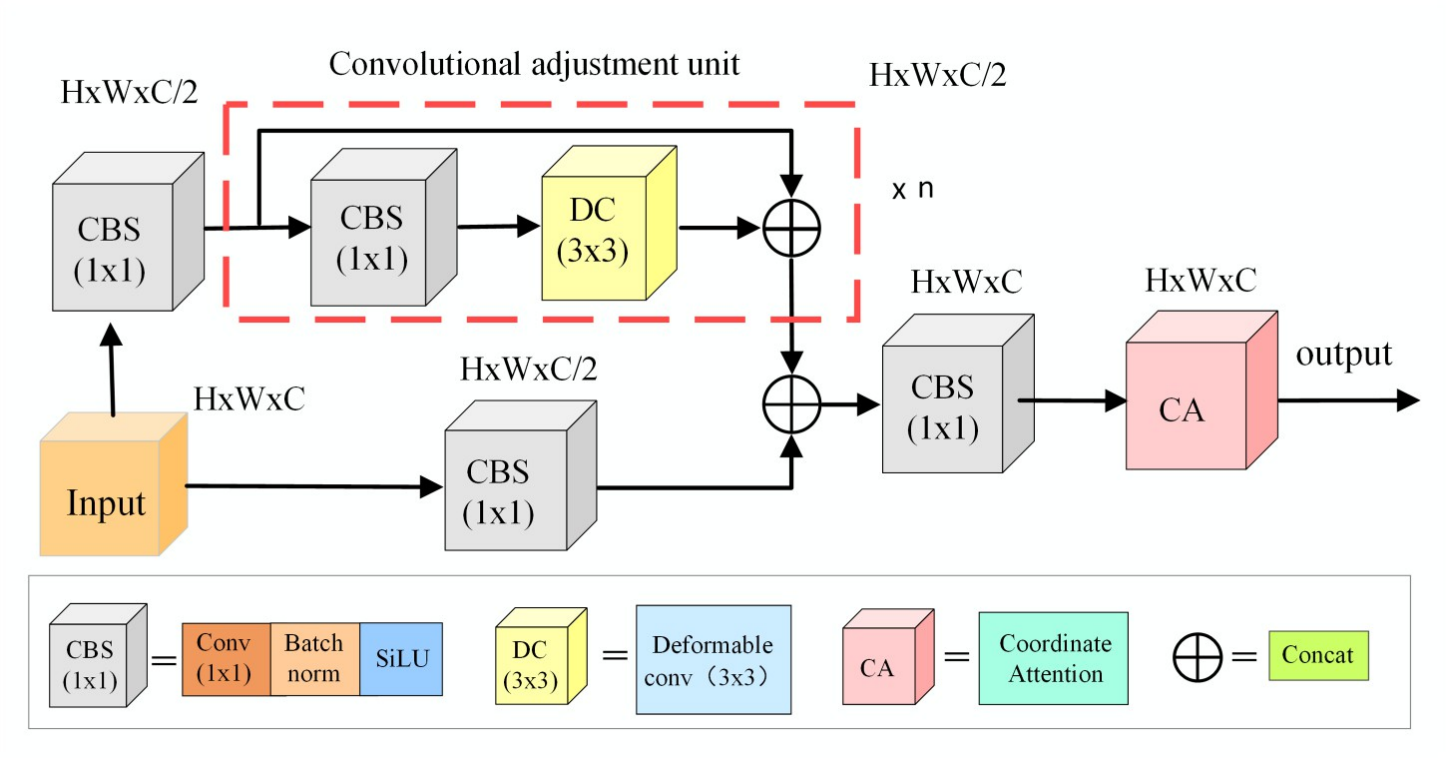
AFEM module structure.

In order to solve the above problems, in this paper, we design an adaptive feature extraction module (AFEM). This module combines the deformable convolution model [21] with the coordinate attention model [22]. The deformable convolution module adaptively learns the offset of each target position in the image to adjust the receptive field of the convolution kernel, so as to obtain the feature information of small targets more effectively. In addition, the coordinate attention module is used to provide spatial and channel dimension attention to small targets in aerial images. This helps to extract more accurate feature information and reduce the interference of complex background. The AFEM module structure is shown in Fig 2.

It is assumed that the feature space of the initial input AFEM module is $G=\left[g_{1},g_{2},\cdots ,g_{c}\right]\in {\mathbb{R}}^{H\times W\times C}$, where *C* is the number of channels of the feature map, *H* and *W* are the height and width of the feature map, respectively. In order to make the model maintain a certain gradient descent, the input feature information is first divided into two branches. After channel transformation, the feature information on the two branches is processed by convolution, batch normalization and SiLU function activation (CBS) to obtain the feature space $S\in {\mathbb{R}}^{H\times W\times C/2}$. The process can be expressed by the formula below:

$$S=\beta \left(E_{1}\left(G\right)\right)$$ (1)

where *E*_1_ is the combination of convolution and batch normalization, β is the nonlinear activation function of SiLU.

After obtaining the feature space *S*, it is fed into the n convolution adjustment units composed of CBS module and deformable convolution module. As the convolution adjustment unit can adaptively adjust the receptive field of the convolution kernel, more feature information of small targets in UAV aerial images can be obtained. In the adjustment unit, the input spatial feature S is fed into the CBS module for processing after the number of channels is halved. The specific processing method is shown in Eq (1). After the CBS module is processed, it is fed into the DC module. The specific processing method of this module is as follows:

$$\text{y}\left(q\right)=\sum_{k=1}^{k}w_{k}\cdot x\left(q+q_{k}+\Delta q_{K}\right)\cdot \Delta m_{k}$$ (2)

where y(*q*) represents the final output feature of *q* point, *x*(*q*) denotes the characteristic of *q*-point input, *w*_*k*_ represents the weight of each pixel in the convolution process, *q*_*k*_ represents the corresponding position of each pixel in the convolution region, *k* is the number of pixels in the convolution region, Δ*q*_*k*_ denotes the offset of each pixel, and Δ*q*_*k*_ ∈ *R*, Δ*m*_*k*_ denotes an adjustable scalar, and Δ*m*_*k*_ ϵ [0,1].

**Fig 3:**
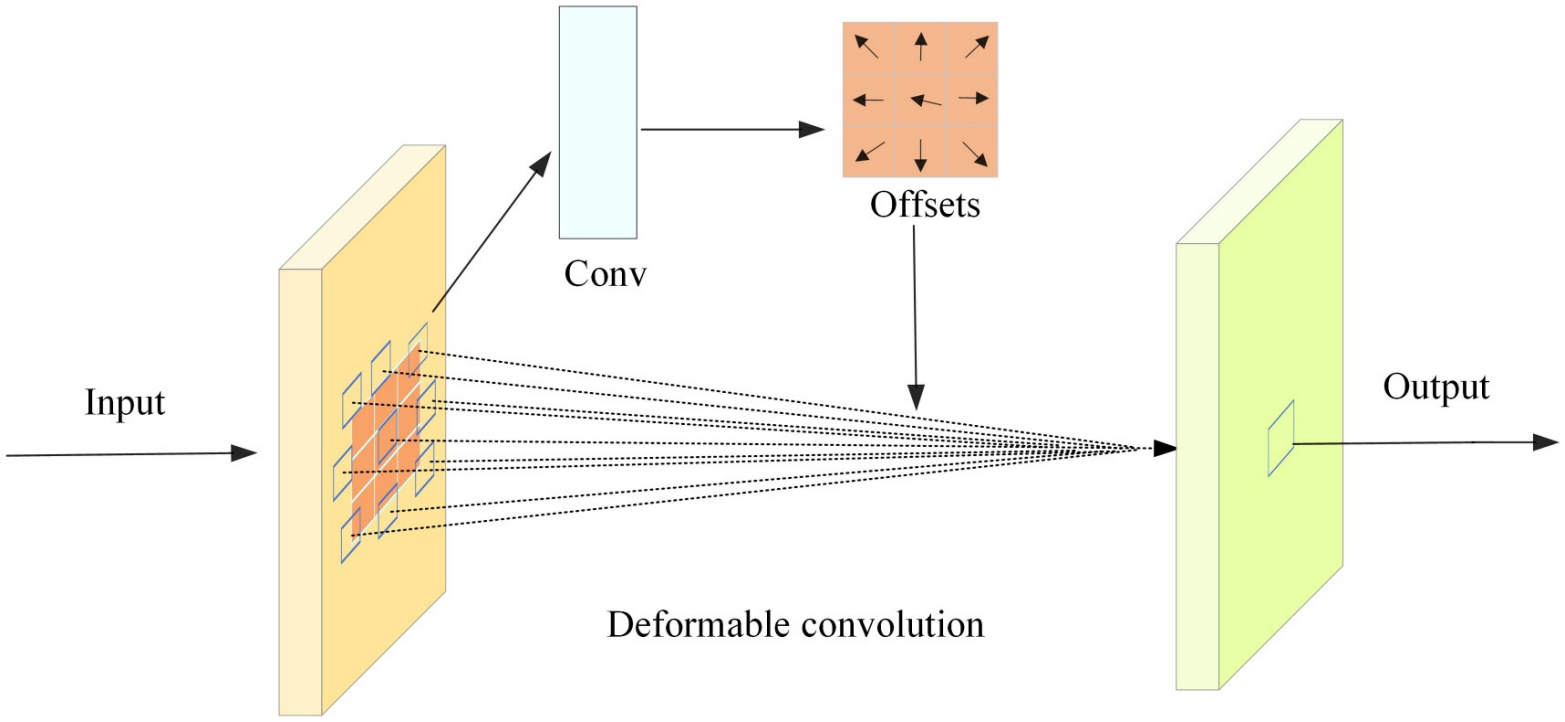
Deformable convolution process.

As shown in Fig 3, the DC module first learns the input UAV aerial image through convolution to obtain the weight *w*_*k*_ and offset Δ*q*_*k*_ corresponding to each image point. Next, the offset position of each pixel in the convolution region is calculated with point *q* as the coordinate origin. Then, the receptive field of the convolution kernel is adjusted according to the calculation results to obtain more feature information of small targets. Finally, the importance of each pixel in the region is judged by the learned adjustment scalar Δ*m*_*k*_, and the feature information is extracted according to the judgment result.

After the DC module extracts the features, the obtained features are concatenated with the features in the initial input adjustment unit as the output of the entire convolution adjustment unit. At the same time, in order to maintain a certain gradient of the model during training and restore the number of channels of the feature, the output of the adjustment unit is concatenated with the feature space *S*, and then fed into the CBS module for processing. The process can be expressed as:

$$X=\beta \left(E_{1}\left(Cat[S,B]\right)\right)$$ (3)

where *Cat* [⋅,⋅]is the feature concatenation operation, *B* is obtained by concatenating the output of the convolution adjustment unit with the feature space *S*.

In order to extract accurate and effective features of small targets, the feature map is then sent to the CA module. Suppose that the feature space of the input feature map is $X=\left[x_{1},x_{2},\cdots ,x_{c}\right]\in {\mathbb{R}}^{C\times H\times W}$. After entering the attention module, the position information of each target in the feature map is first encoded. In order to prevent the phenomenon that the position information is difficult to save, two one-dimensional pooling kernels (*H*,1) and (1, *W*) are used to encode the coordinate information of each target in the map along the horizontal and vertical directions respectively. The specific ways of encoding are as follows:

$$P_{C}=\frac{1}{H\times W}\sum_{i=1}^{H}\sum_{j=1}^{W}x_{c}\left(i,j\right)$$ (4)

Where *i* denotes the vertical height of the feature map, *j* denotes the horizontal width of the feature map, *P*_*c*_ represents the output of channel *C* after encoding.

From the above formula (4), the horizontal position coding information $P_{c}^{\text{w}}$ and the vertical position coding information $P_{c}^{h}$ of each target in the aerial image can be obtained respectively:

$$P_{c}^{h}\left(h\right)=\frac{1}{W}\sum_{0\leq i<W}x_{c}\left(h,i\right)$$ (5)

$$P_{c}^{\text{w}}\left(\text{w}\right)=\frac{1}{H}\sum_{0\leq j<H}x_{c}\left(j,w\right)$$ (6)

where *h* represents the specific height of the target in the feature map, *w* represents the specific width of the target in the feature map.

After obtaining the position coding information of each target in the vertical and horizontal directions of the aerial image, the horizontal and vertical information is integrated by concatenation. Then, the obtained feature maps are subjected to convolution operation, batch normalization processing and nonlinear activation to complete the conversion of position coding information. Finally, the dimension of the feature map is adjusted by the reduction coefficient *r* to reduce the computational complexity of the network. it can be expressed as:

$$f=\delta \left(F_{1}\left(\text{Cat}\left[P^{h},P^{w}\right]\right)\right)$$ (7)

Where *F*_1_ is a combination operation of convolution and batch normalization, δ is the *h-swish* nonlinear activation function.

It is assumed that the feature space of the vertical dimension after position information coding is $f\in {\mathbb{R}}^{C\times H\times 1}$, and the feature space of the horizontal dimension is $f\in {\mathbb{R}}^{C\times W\times 1}$. After the above conversion, the feature space of the newly obtained feature map becomes $f\in {\mathbb{R}}^{C/r\times \left(H\times W\right)}$.

In order to obtain the horizontal and vertical feature attention weights, the transformed feature space needs to be decomposed again. After splitting the feature space $f\in {\mathbb{R}}^{C/r\times \left(H\times W\right)}$ of the feature map in the horizontal and vertical directions, the independent feature spaces $f^{\text{w}}\in {\mathbb{R}}^{C/r\times W}$ and $f^{h}\in {\mathbb{R}}^{C/r\times H}$ in the horizontal and vertical directions are obtained respectively. Next, the 1x1 convolution is used to extract the feature information of the two dimensions and restore the dimension of the feature map to the size of *H* ×*W* × *C*, so as to facilitate the subsequent weight conversion. Then, the *Sigmoid* function is used for activation processing to obtain the output *g*^*h*^ and *g*^*w*^. The process can be expressed as:

$$g^{h}=\alpha \left(F_{h}\left(f^{h}\right)\right)$$ (8)

$$g^{w}=\alpha \left(F_{w}\left(f^{w}\right)\right)$$ (9)

Where α represents the *Sigmoid* activation function, *F*_*h*_ represents the convolution transform in the vertical direction, and *F*_*w*_ represents the convolution transform in the horizontal direction.

After obtaining the above outputs *g*^*h*^ and *g*^*w*^, it is converted into a weight matrix *y*_*c*_(*i*, *j*) by the following method.


$$y_{c}\left(i,j\right)=x_{c}\left(i,j\right)\times g_{c}^{h}\left(i\right)\times g_{c}^{w}\left(j\right)$$ (10)

**Fig 4:**
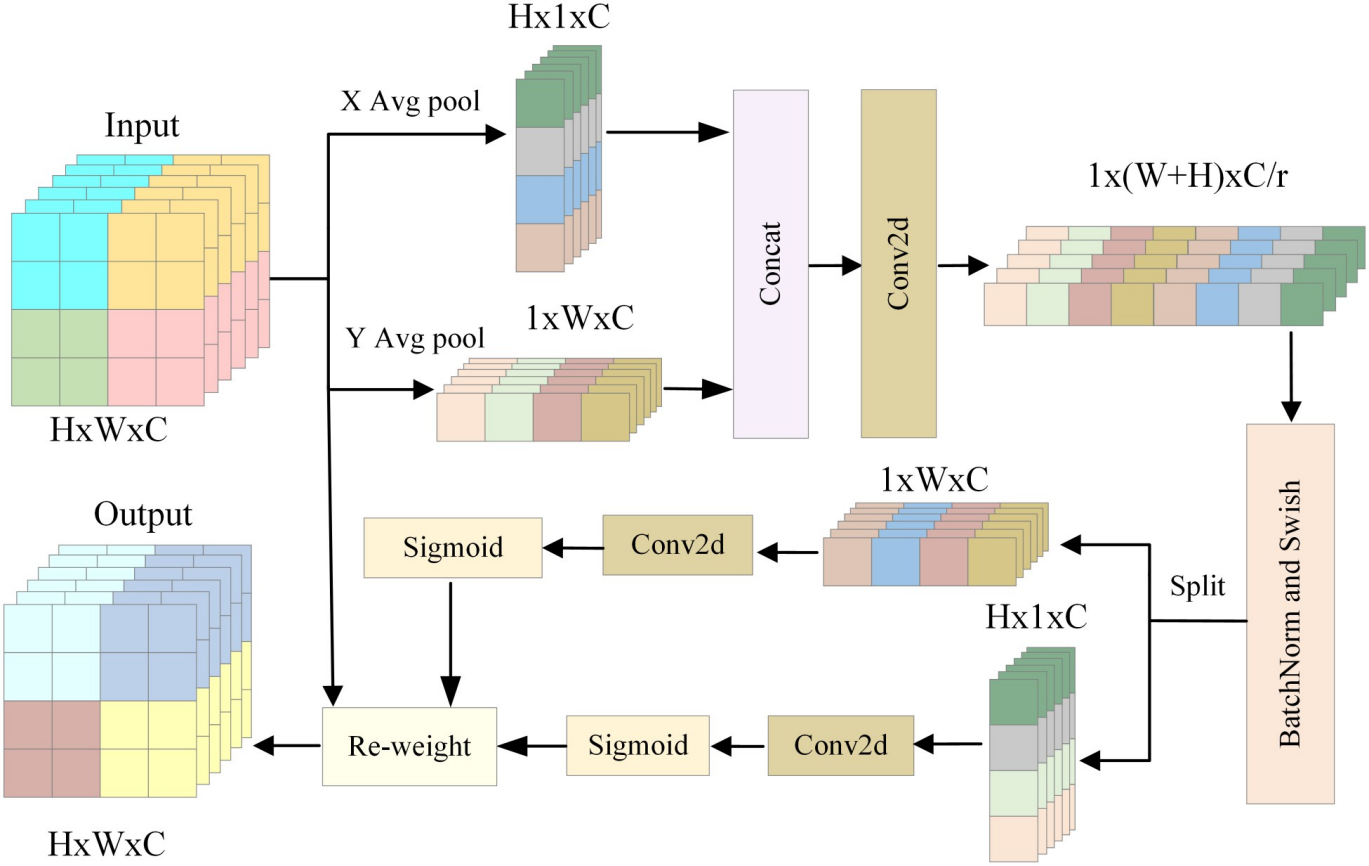
Feature extraction process of CA module.

After the above operations, the input feature space is finally transformed from $X=\left[x_{1},x_{2},\cdots ,x_{c}\right]\in {\mathbb{R}}^{C\times H\times W}$ to $Y=\left[y_{1},y_{2},\cdots ,y_{c}\right]\in {\mathbb{R}}^{C\times H\times W}$. It contains channel information, vertical and horizontal spatial information, thereby completing the accurate and effective extraction of aerial image feature information and obtaining the final output of the AFEM module. The specific extraction process of the CA module feature is shown in Fig 4.

Through the interaction between the different modules designed above, AFEM can adaptively adjust the receptive field of the convolution kernel to obtain more feature information of small target objects in the image. At the same time, it can pay attention to the input UAV aerial image in the channel, vertical and horizontal space. This enables the network to quickly locate the spatial position of small targets in the image for accurate and effective feature extraction. Due to the above functions, the AFEM module further enhances the network’s ability to detect small targets in UAV aerial images.

### 3.3. Adaptive feature fusion network

**Fig 5:**
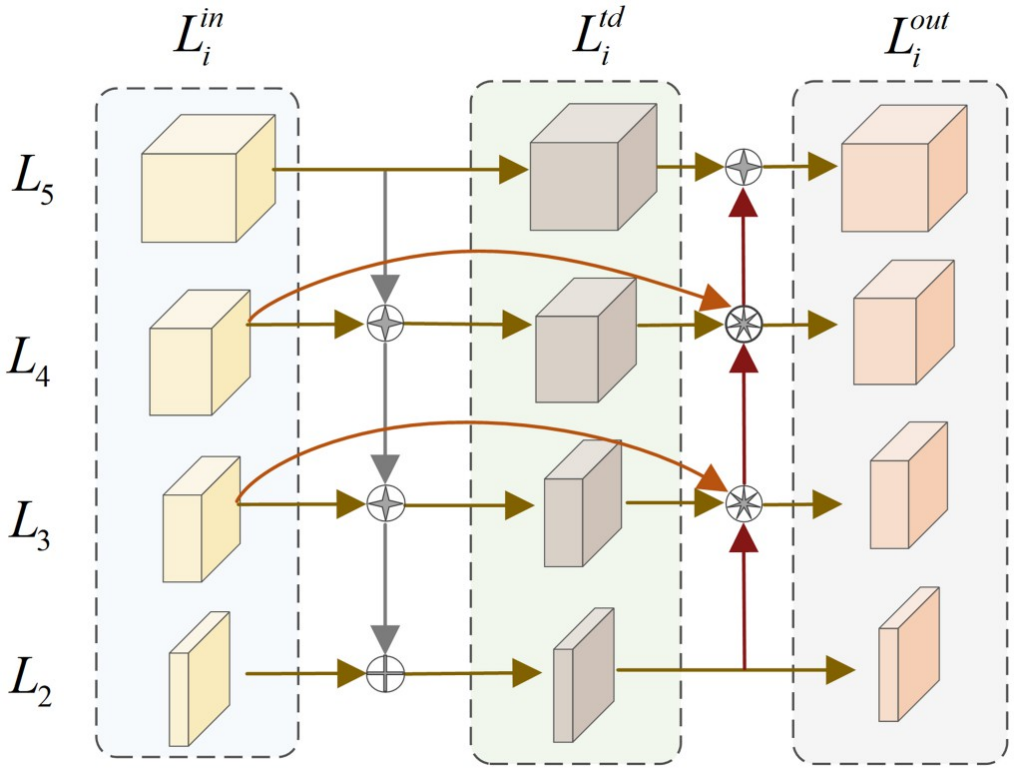
SBiFPN network structure.

In the process of feature extraction, shallow features can describe the location, texture, and other information of the detected target, while deep features are more likely to express the semantic information of the detected target. As small target objects are small in the image, the shallow feature information is more conducive for their characterization. However, with the increase of network depth, the shallow feature information of small target objects in aerial images becomes gradually weakened. In order to prevent the loss of shallow feature information, so that the network can better fuse the feature information of small targets in the image, we design a new feature fusion network based on EfficientDet [23], for the shallow feature information of more small targets to be transmitted backward. The structure of the network is shown in Fig 5.

The previous network was designed by combining FPN [24] and PANet [25] to fuse the feature information. But, the structure fuses the feature information of the same scale indiscriminately, which weakens the shallow feature information in the aerial image. Therefore, we design a new feature fusion network structure SBiFPN (Simplify Bidirectional Feature Pyramid Network) to fuse the feature information extracted by the backbone network. It is assumed that the feature set obtained by feature extraction of aerial images is ${\vec{L}}^{in}=\left(L_{1}^{in},L_{2}^{in},\cdots L_{i}^{in}\right)$, where $L_{i}^{in}$ denotes the input feature of the *i*-th level obtained by the original image after 2^*i*^ times of downsampling. Through continuous convolution in the backbone network, five different levels of feature information can be extracted. Because the network has less feature information extraction for the first level, this paper only fuses the features of the four effective levels of.*L*_2_ ~ *L*_5_.

Since the feature information of different scales has different importance for the detected target, in order to strengthen the detection of small target objects, the feature information of certain scales should be emphasized in feature fusion. After the feature information is input into the fusion network, the fusion method of feature information at different scales can be expressed as:

$$N=\sum_{i}\frac{w_{i}}{\partial +\sum_{j}w_{j}}\cdot I_{i}$$ (11)

Where *N* is the fused feature information, *I*_*i*_ is the input feature information, *∂* is a constant that prevents data fluctuations and takes 0.0001, *w*_*i*_ and *w*_*j*_ are learnable weights that can be automatically learned and adjusted during training to achieve optimality.

For example, through formula (11), the feature fusion of *P*_4_ can be expressed as:

$$L_{4}^{td}=Conv\left(\frac{\text{Cat[}w_{1}\cdot L_{4}^{in},w_{2}\cdot Resize\left(L_{5}^{in}\right)\text{]}}{w_{1}+w_{2}+\partial }\right)$$ (12)

$$L_{4}^{out}=Conv\left(\frac{\text{Cat}\left[w_{1}^{\prime }\cdot L_{4}^{in},w_{2}^{\prime }\cdot L_{4}^{td},w_{3}^{\prime }\cdot Resize\left(L_{3}^{out}\right)\right]}{w_{1}^{\prime }+w_{2}^{\prime }+w_{3}^{\prime }+\partial }\right)$$ (13)

Where *Conv*(⋅) represents the convolution operation, *Resize*(⋅) represents upsampling or downsampling operation, $L_{4}^{td}$ denotes the top-down intermediate feature in the fourth level, $L_{4}^{out}$ represents the output of the bottom-up fourth-level feature.

In the three different scales of *L*_3_ ~ *L*_5_, the features are also fused in the above ways. Because the shallow feature information contained in the *L*_2_ level is more, we use vector direct concatenation to fuse it in order to transmit it as much as possible.

Due to the large number of small targets in UAV aerial images, it is necessary for the network to obtain rich shallow feature information for better detection. Through the adaptive feature fusion network designed in this paper, more shallow feature information can be transmitted backward. It effectively avoids the loss of feature information such as small target location and texture when performing UAV aerial image detection, thereby improving the detection ability of the network.

### 3.4. Multi-scale detection network

**Fig 6:**
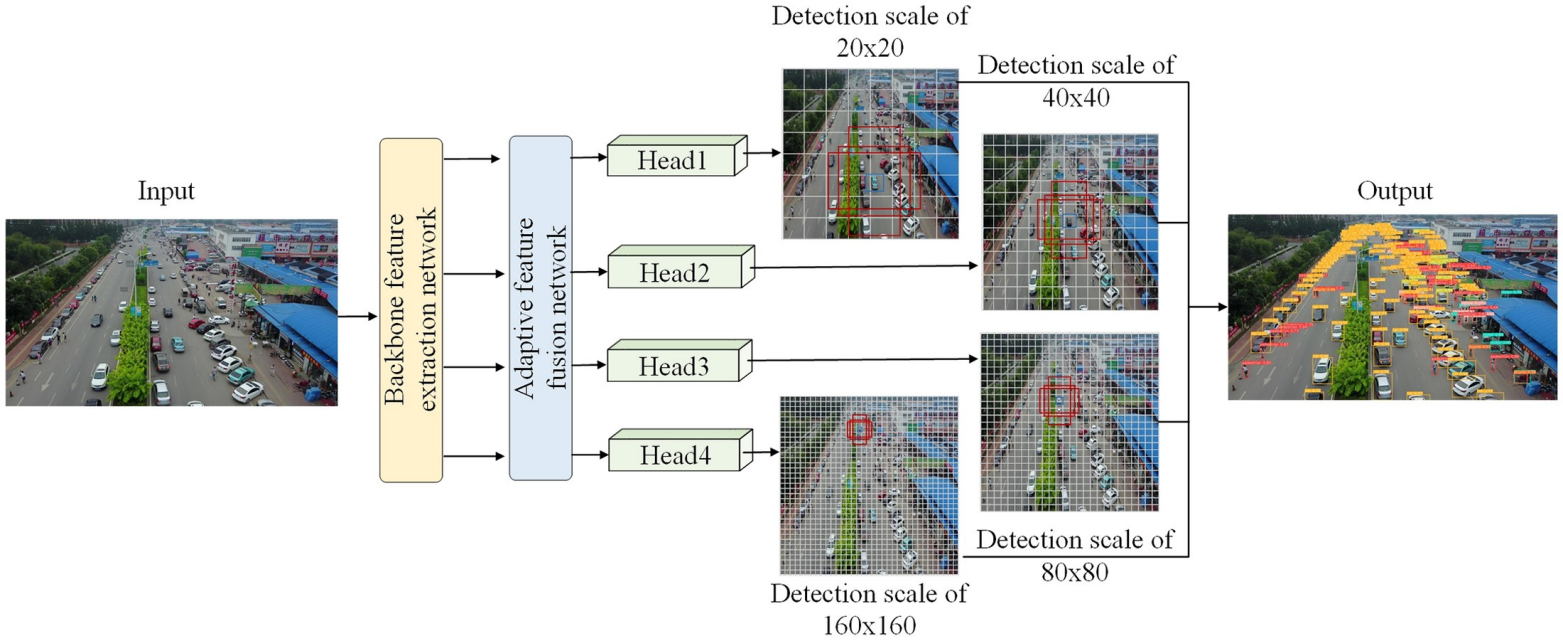
Multi-scale network detection process.

In the image target detection task, there are often multiple types of detected targets. Due to the size of each target, shooting angle, shooting height and other reasons, the scale of each target in the image will change accordingly. At this time, the network needs to have the ability to perform multi-scale detection. When detecting each target in the aerial image, there are many small-scale targets and the scale of each target changes greatly. Therefore, in order to adapt to the detection of each target in the UAV aerial image, an additional small target detection head is added on the basis of the benchmark network after analysis. Finally, the detection scale of the network is changed to four, and the ability of UAV aerial images to detect small targets is further strengthened. The designed multi-scale network detection process is shown in Fig 6.

Due to the high resolution of the feature map obtained after sampling at low magnification, it can carry more fine-grained information of small target objects, and the receptive field of the network is also larger. Therefore, in this work, we fuse the feature map obtained by 4 times downsampling in the feature extraction network with the feature map obtained by 8 times upsampling in the adaptive feature fusion network. Then, we input the output feature map into the fourth detection head for target prediction. In this way, on the one hand, the shallow feature information obtained by the network can be fully utilized, and on the other hand, the perception ability of the network for the feature map can be further improved. Therefore, the increased detection scale enables the network to detect small-scale targets in the image more accurately.

The newly designed detection network can generate four feature maps of different scales for the detected targets in the UAV aerial image. Through the division of labor and coordination between each feature map, the network can easily cope with the detection tasks of different targets in the image, and the detection effect for small-scale targets is very obvious.

## 4. Experiment and discussion

### 4.1. Experimental dataset

On the data set, this study selects the representative UAV target detection public data set VisDrone2019 [26]. The data set is cut from videos taken by drones at different heights. It contains a total of 10209 images of 1360x765 and 960x540 sizes. Through random division, 6471 is used as the training set, 3190 as the test set, and the remaining 548 as the verification set. After a series of processing and labeling, the final data set contains 10 categories of pedestrians, people, cars, trucks, buses, trucks, motorcycles, bicycles, awning tricycles, and tricycles, with a total of about 2.6 million instance target samples.

### 4.2. Experimental platform and parameter settings

The experimental platform of this paper adopts Windows10 system, the processor is Intel i5-10400 2.90 GHz, the image processor is NVIDIA GeForce RTX3060, and the deep learning framework is Pytorch. During the training, the initial learning rate of the network model is set to 0.01, the size of the input image is 960x960, the momentum factor is set to 0.937, the weight decay parameter of the optimizer is set to 0.0005, and the intersection over the union (IoU) threshold of the real and prediction frames is at 0.5. Considering the memory size of the GPU, the batch size of each round is 10. After all the network parameters are set, a total of 100 epoch trainings are performed on each model.

### 4.3. Ablation experiment

In order to verify the effectiveness of the designed network structures in the target detection task of UAV aerial images, in this work, we take YOLOv5s as the baseline network and conduct a series of ablation experiments on the VisDrone dataset. By combining the requirements of the UAV aerial image target detection task in the actual application process, we select the model size, parameters, floating point operations per second (FLOPs), frames per second (FPS), average precision (AP) and mean average precision (mAP) to evaluate the detection effect of the model. The results of the experiment are shown in Table 1.

**Table 1:** Ablation experimental results on visdrone.

	Method			mAP	Modle Size	Parameters	FLOPs	FPS
	AFEM	SBiFPN	Multi-Scale					
**Baseline**				36.5	13.89MB	7.04M	15.9G	36.8
	**✓**			36.9	14.26MB	7.22M	14.3G	36.1
		**✓**		36.8	14.08MB	7.14M	16.1G	35.9
			**✓**	37.4	15.03MB	7.19M	18.8G	28.6
	**✓**	**✓**		37.0	14.44MB	7.32M	14.7G	35.8
	**✓**		**✓**	37.9	15.40MB	7.38M	17.3G	29.3
		**✓**	**✓**	38.1	15.17MB	7.26M	19.1G	27.2
	**✓**	**✓**	**✓**	38.5	15.56MB	7.46M	17.7G	26.9

From the experimental results, it can be seen that after adding the AFEM module to the baseline network, the size of the model is 14.26 MB, the number of parameters is 7.22 M (1M = 10^6^), the amount of floating-point computation is 14.3 G, the detection speed is 36.1 frames/s, and the mean average accuracy reaches 36.9 mAP. The above experimental results also fully prove the effectiveness of the AFEM module. Although the addition of the AFEM module to the baseline network resulted in an increase in the size of the network model and the number of parameters by 0.37 MB and 0.18 M, respectively, however, due to the coordinated attention model in AFEM being able to provide spatial and channel attention to various targets in drone aerial images, thereby eliminating redundant backgrounds in the images, the network’s floating-point computation is reduced by 1.6G. In addition, since the deformable convolutional unit in the AFEM module can adaptively adjust the size of the convolutional kernel’s receptive field according to each target in the image, it makes the network extract more small target feature information, and the detection accuracy is improved by 0.4 percentage points. From the perspective of detection speed, there is no significant change after adding the AFEM module, and compared to the baseline network, it only reduces by 0.7 frames/s.

After using the SBiFPN feature fusion network on the basis of the baseline network, the model size of the network is 14.08MB, the parameter count is 7.14M, and the floating-point computation is 16.1G. Due to the addition of the SBiFPN network structure, more shallow features containing small target information are transmitted backward. In addition, the corresponding calculations become more complex in the adaptive fusion process of shallow and deep features. The above reasons ultimately led to an increase of 0.1M and 0.2G in network parameter and computational complexity, respectively. Although the network’s parameter and computational complexity slightly increased, it also allowed the network to obtain more small target feature information, with an average accuracy improvement of 0.3 percentage points, reaching 36.8%. Since the SBiFPN network structure does not change the functional modules of the original network too much, the detection speed is reduced by only 0.9 frames/s.

In order to verify the performance of the multi-scale detection network, relevant experiments were carried out on the baseline network. The results show that after the introduction of the multiscale detection network designed in this paper, although the increase of functional modules makes the size of the network model, the number of parameters, and the amount of computation increase by 1.14MB, 0.15M, and 2.9G, respectively, and the speed of the detection decreases by 8.2 frames/s. It is able to allow the network to obtain a wider sensory field, which improves the mAP accuracy of the network model by 0.9 percentage points, reaching 37.4%. This shows that the multi-scale detection network used in this paper can significantly improve the model’s ability to detect small targets in UAV aerial images.

Finally, after adding the above three structures in turn, the parameters and calculation amount of the network model slightly increase by 0.42 M and 1.8 G, respectively. The mAP accuracy value of UAV aerial image target detection has a more obvious improvement effect, which is 2.0 percentage points higher than that of the original network. Although the detection speed of the model is reduced compared to the original network, it still reaches 26.9 frames/s, which can better meet the requirements of real-time detection. Therefore, from the above results, the UAV-YOLO detection method proposed in this paper can better detect the target of UAV aerial image, and the model is only 15.56 MB in size, which is easy to transplant and deploy. Benefiting from the above advantages, UAV-YOLO is very suitable for UAV aerial image target detection tasks.

### 4.4. Comparing the detection effect of UAV-YOLO network with the original network model

**Fig 7:**
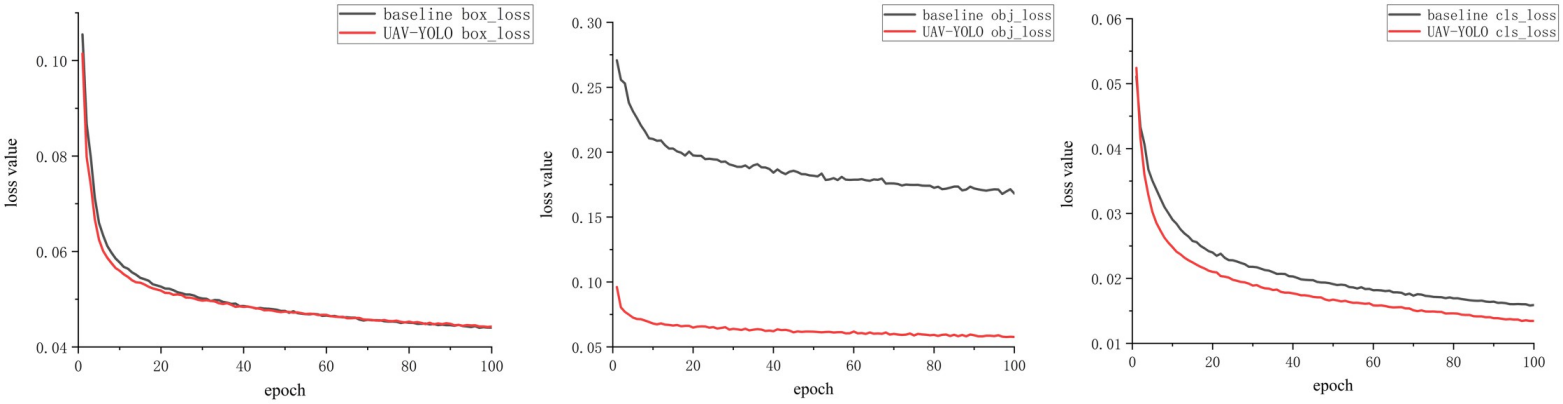
Comparison of training loss between UAV-YOLO and baseline methods: (a): Box loss comparison; (b): Objectness loss comparison; (c): Classification loss comparison.

In order to verify the performance difference between the UAV-YOLO network and the baseline network in processing the target detection task of UAV aerial images, based on the VisDrone data set, we train the two detection methods respectively. The parameter settings during training are the same as those in Section 4.2. After 100 epochs of training, the loss values of the two network models basically reached the convergence state. The training loss of the UAV-YOLO network and the baseline network is shown in Fig 7.

**Fig 8:**
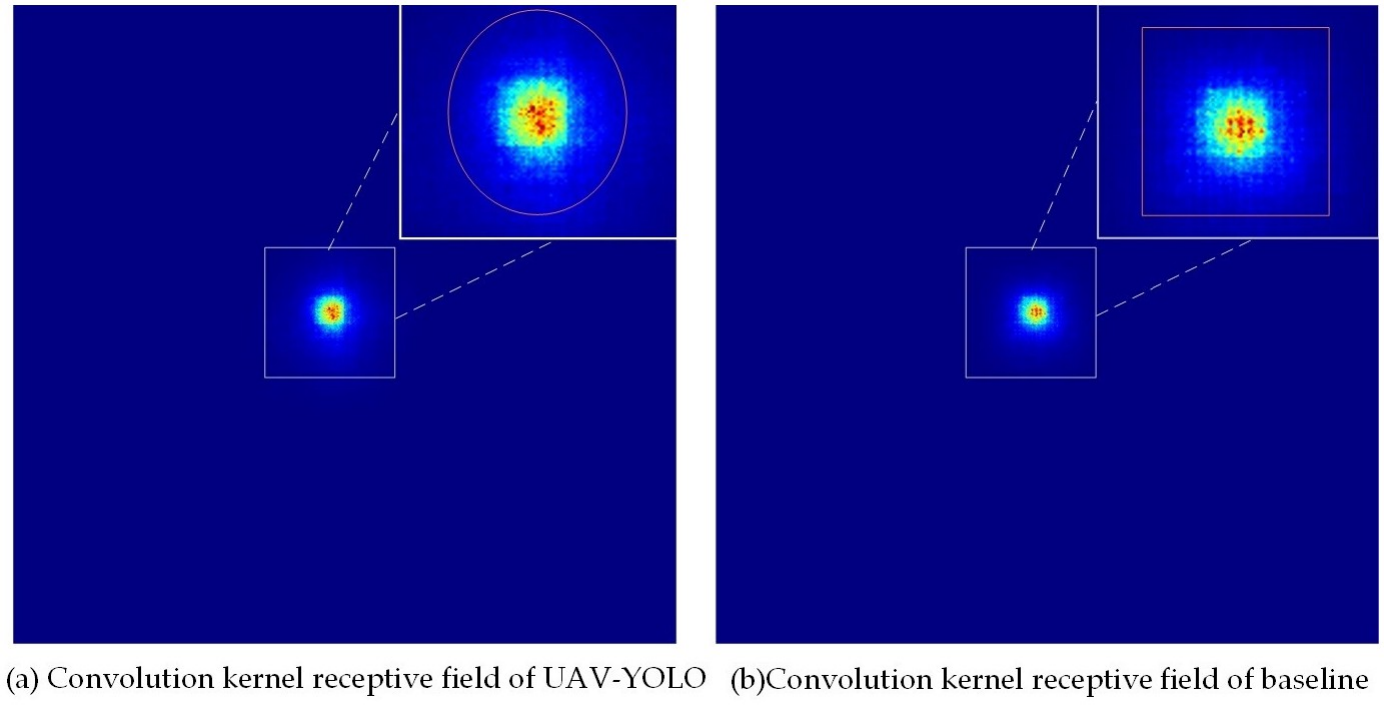
Comparison of receptive fields of the two network convolution kernels.

From the comparison chart of the loss values during the training process of the two networks, it can be seen that compared to the baseline network, the UAV-YOLO’s various loss values decrease faster, making it easier for the network to reach a convergence state. After the training is completed, the images with more small targets in the VisDrone test set are used to test the two network models respectively, and the convolution kernel receptive field in the network detection process is visualized. From the visual in Fig 8, it can be seen that after adding the AFEM module, the UAV-YOLO has a significant change in the shape of the receptive field area of each target in the image, and the area becomes brighter. This phenomenon shows that the AFEM module enables the network to obtain more effective receptive fields. For the baseline network, its convolution kernel can only provide the receptive field of the square area, but UAV-YOLO can automatically adjust the shape area of the receptive field of the convolution kernel according to the detected target. The adjusted receptive field can better adapt to the detection of the target, and can provide a more effective receptive field. This enables the network to obtain more feature information of small targets in the image.

**Fig 9:**
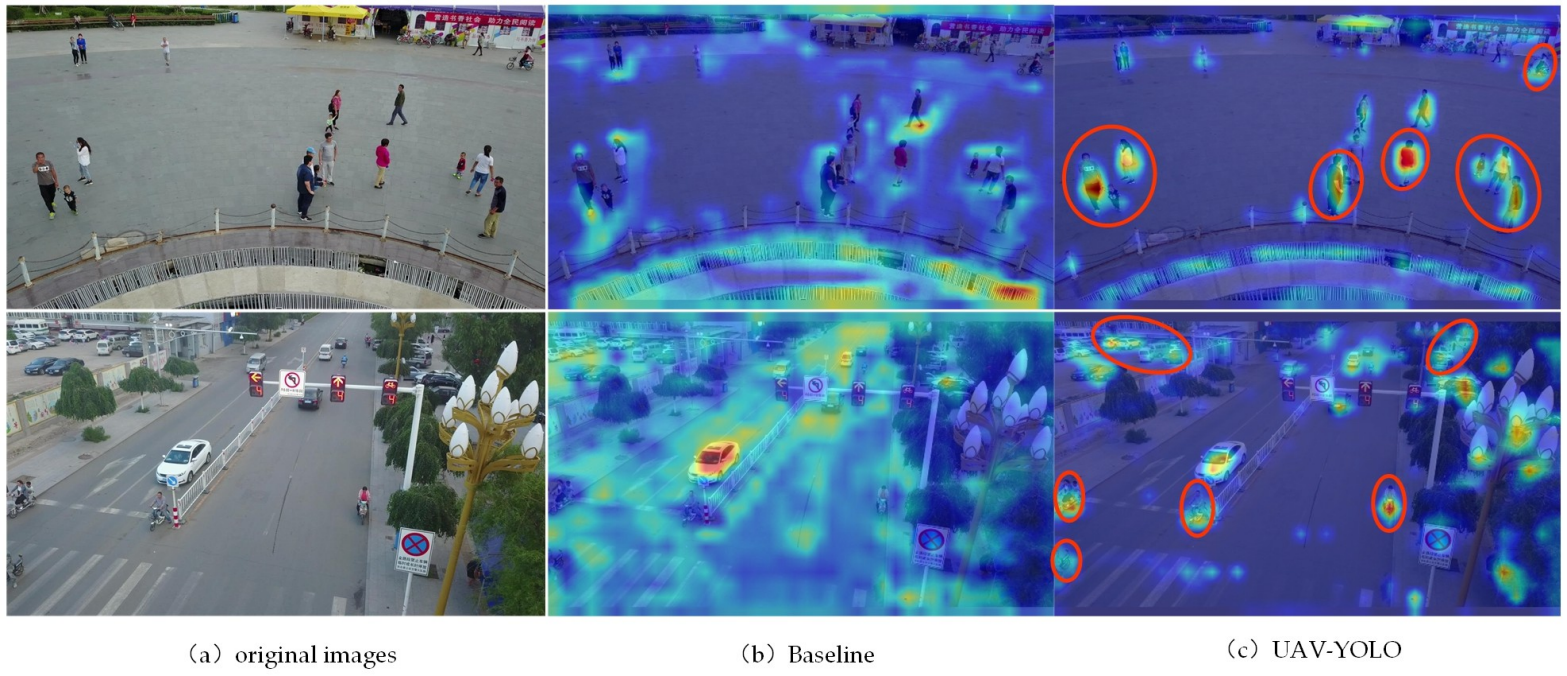
Visualization of the focus areas of the two networks during the detection process.

We also visualize the key focus areas of the two networks in the target detection process, and find out the performance differences between the two networks through analysis and comparison. From the comparison between (a) and (b) in Fig 9, it can be seen that after adding the AFEM module, UAV-YOLO can effectively reduce the redundant background in the image and provide focus on the detected target, enabling the network to obtain more accurate and effective feature information. This further reduces the calculation amount of the network in the detection process.

**Fig 10:**
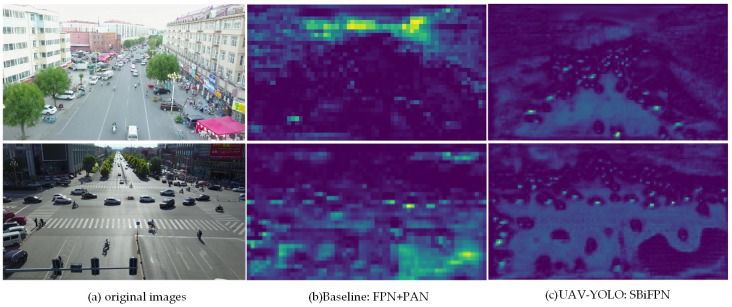
Visualization of feature maps in two network detection processes.

In order to compare the difference of feature fusion between the two detection networks, we visualize the UAV aerial image feature maps obtained by the two networks during the target detection task. From the visualization result in Fig 10, it can be seen that after the feature fusion of the baseline network, the shallow feature information such as the shape and texture of the various types of small target objects in the image are gradually lost. However, after using the SBiFPN feature fusion network, the shallow feature information of each target in the feature map is retained. Therefore, compared to the baseline, UAV-YOLO is more friendly for the detection of small target objects and can be more suitable for the target detection task of UAV aerial images.

**Fig 11:**
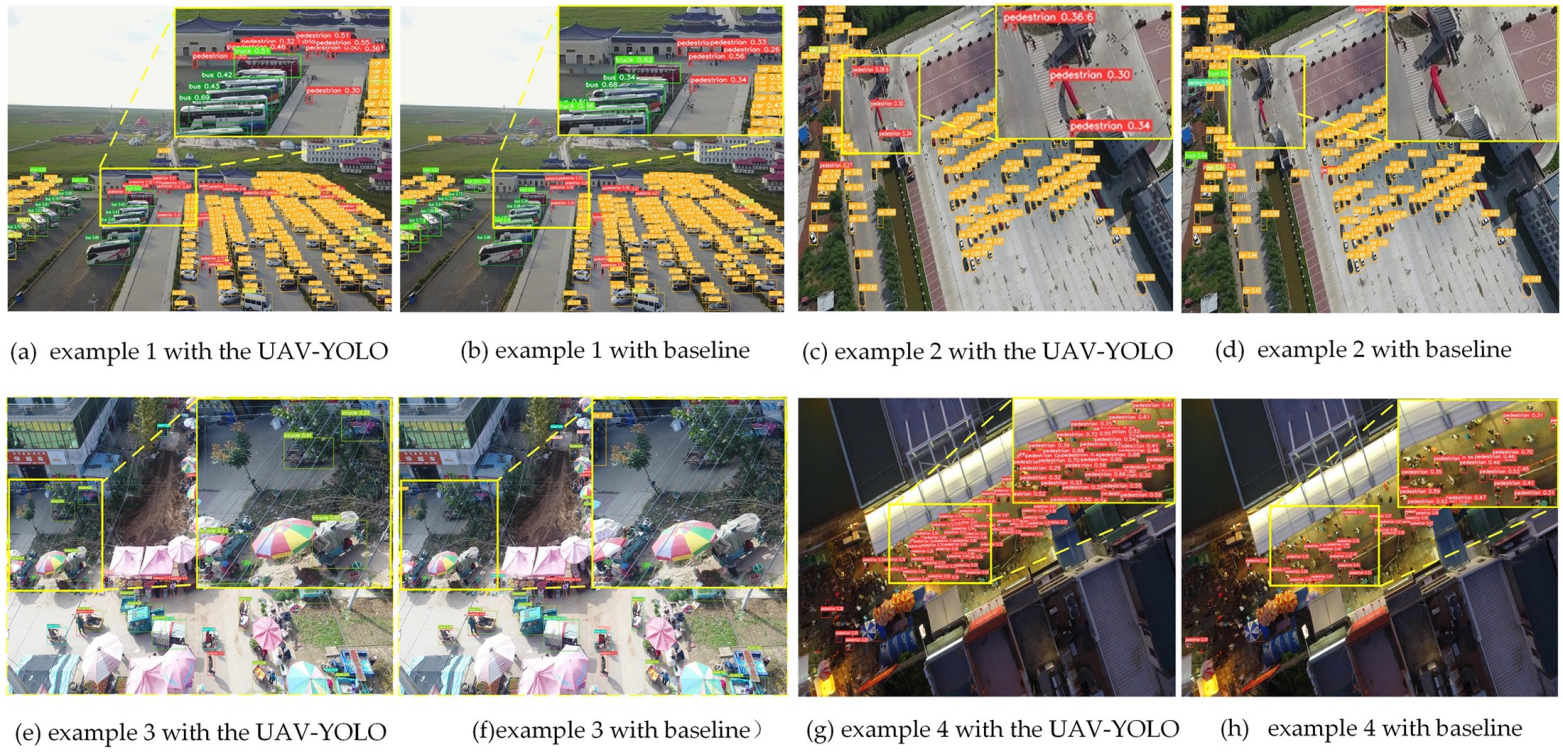
The comparison of the detection results of the two methods in different scenarios.

Finally, in the VisDrone test set, we select the images under the typical scenes of multi-scale, high altitude, complex background and illumination influence to compare the actual detection effects of the two methods. The relevant experimental results are shown in Fig 11.

By comparing the detection results of Fig 11(a) and 11(b), it can be seen that in the scene with large scale changes, the baseline network has more pedestrians that have not been successfully detected, while UAV-YOLO achieves good detection of small targets due to the multi-scale detection network. In addition, from the comparison between Fig 11(c) and 11(d), it can be seen that in the high-altitude scene, the baseline network cannot detect small targets, while UAV-YOLO can obtain more abundant shallow feature information of small targets and detect more small target objects. Through the comparison of Fig 11(e) and 11(f), it can be seen that in complex backgrounds, UAV-YOLO can provide attention to the detected targets through the AFEM module in the backbone network, and extract more accurate and effective feature information. Thus, it can detect more targets that are ignored by the baseline network. Finally, through the comparison of Fig 11(g) and 11(h), it can be found that under the condition of insufficient illumination at night, UAV-YOLO also shows better detection ability than the baseline network.

After the detection, we counted the detection results of the above image examples. The related results are shown in Table 2. From the statistical results, it can be seen that in three environments with large scale changes, high-altitude, and complex background, UAV-YOLO shows better detection results compared to the baseline method, leading the baseline method by 4.1, 3.4, and 2.2 percentage points, respectively. In addition, in the case of insufficient illumination at night, UAV-YOLO even shows a significant lead over the baseline method, reaching 36.1 percentage points.

**Table 2:** Statistics of the detection results of the two methods on the above image examples.

Image examples	Number of targets	Number of targets detected		Detection ratio	
		baseline	UAV-YOLO	baseline	UAV-YOLO
1	335	285	299	85.1	89.2
2	120	97	101	80.8	84.2
3	180	145	149	80.6	82.8
4	169	44	105	26.0	62.1

Through the comparative analysis of the above detection results, it can be found that in a variety of typical UAV aerial scenes, the baseline network is prone to missed detection and false detection, while UAV-YOLO can achieve better detection results. Thus, UAV-YOLO can better meet the target detection tasks requirements of UAV aerial image.

### 4.5. Comparison of different models

In order to make a more comprehensive evaluation of the proposed UAV aerial image target detection method, we compare the detection performance of UAV-YOLO with the current mainstream UAV aerial image target detection method. The performance evaluation indexes used are AP and mAP of each target category. The detection results of each method on the VisDrone test set are shown in Table 3.

**Table 3:** The results of different types of detection methods on the VisDrone test set.

Method	Target categories										mAP
	Pedestrian	People	Bicycle	Car	Van	Truck	Tri	Awn-tri	Bus	Motor	
FA_CSKD [20]	10.5	7.5	2.6	47.7	22.2	16.9	8.6	4.0	28.8	12.7	16.2
SAMFR-Cascade RCNN [8]	34.46	23.12	**21.27**	59.96	40.72	30.32	26.48	13.12	47.47	31.35	32.8
DMNet [27]	28.5	20.4	15.9	56.8	37.9	30.1	22.6	14.0	47.1	29.2	30.3
CDNet [27]	35.6	19.2	13.8	55.8	**42.1**	38.2	**33.0**	**25.4**	49.5	29.3	34.2
Cascade R-CNN [28]	22.6	14.8	7.6	54.6	31.5	21.6	14.8	8.6	34.9	21.4	23.2
DBAI-Det [26]	36.7	12.8	14.7	47.4	38.0	41.4	23.4	16.9	31.9	16.6	28.0
CenterNet [29]	22.6	20.6	14.6	59.7	24.0	21.3	20.1	17.4	37.9	23.7	26.2
YOLOv3-LIFT [30]	34.5	**23.4**	7.9	70.8	31.3	21.9	15.3	6.2	40.9	32.7	28.5
YOLOv4 [18]	24.8	12.6	8.6	64.3	22.4	22.7	11.4	7.6	44.3	21.7	30.7
MSA-YOLO [31]	33.4	17.3	11.2	76.8	41.5	41.4	14.8	18.4	60.9	31.0	34.7
YOLOv7-tiny [32]	30.3	21.0	11.5	75.0	38.2	37.9	17.8	17.9	56.2	33.0	33.9
UAV-YOLO	**39.4**	22.9	16.4	**79.8**	**42.1**	**45.3**	20.5	20.7	**61.2**	**36.8**	**38.5**

The best detector is bold.

From the test results in Table 3, it can be seen that compared with other types of detection methods, the algorithm model UAV-YOLO proposed in this paper can better detect each target in the UAV aerial image, and achieves the best detection results in the categories of pedestrian, car, van, truck, bus, and motor. The AP values of the six categories are 39.4%, 79.8%, 42.1%, 45.3%, 61.2%, and 36.8%, respectively. In addition, although the method proposed in this paper does not reach the optimal level in the categories of people, awning tricycle and tricycle, the detection effect for these three categories is still at the upper-middle level. Finally, for the comprehensive performance of the network, by comparing with other detection methods in Table 3, the proposed method achieves 38.5% of the best mAP value on the VisDrone test set.

From the above test results, it can be seen that UAV-YOLO has significant advantages in the target detection task of UAV aerial images. It can fully mine the feature information of small targets in the image and better complete the detection of various types of targets.

### 4.6. Practical application effect test

**Fig 12:**
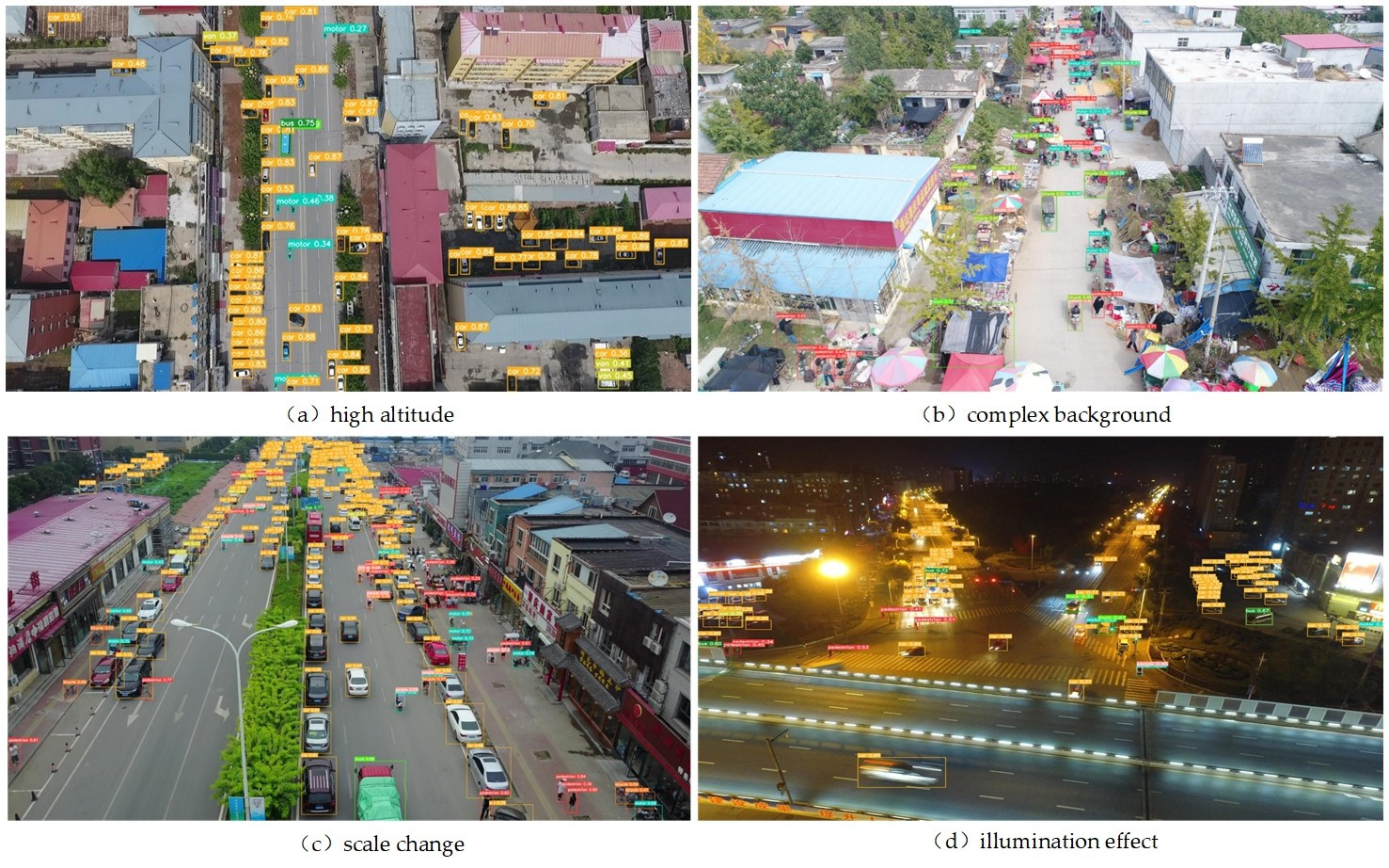
Partial detection results on the VisDrone challenge test set.

In order to verify the performance of UAV-YOLO in practical applications, the target detection challenge test set of UAV aerial image published by VisDrone official website is selected to test the application of the detection method proposed in this paper. Some samples are shown in Fig 12. It can be seen from the figure that UAV-YOLO can still accurately mine the feature information of small target objects in the image under the typical scenarios of complex background, insufficient illumination, high altitude and large target scale change. It better detects each target and the model shows good stability.

**Fig 13:**
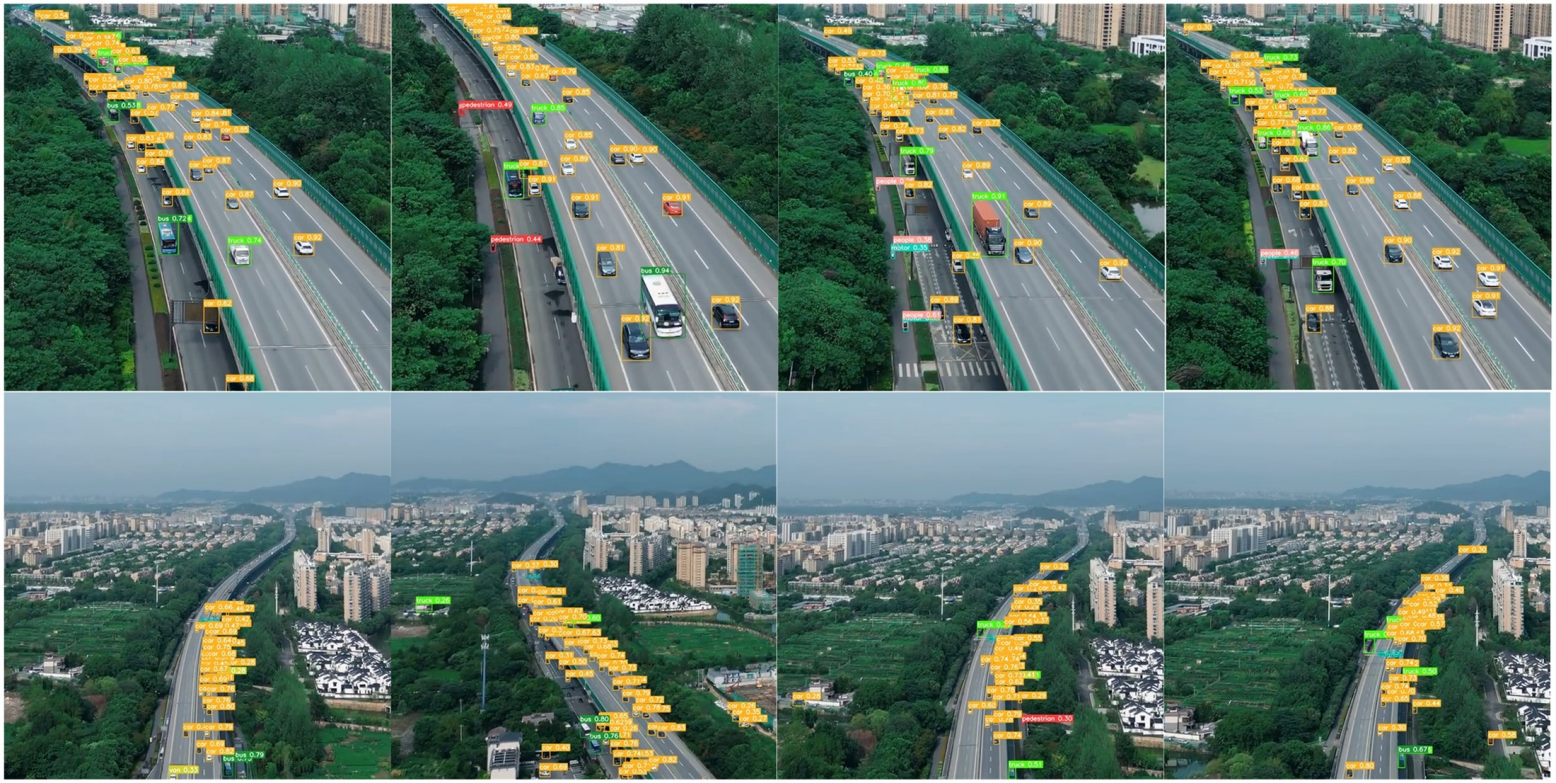
Detection effect in real scenarios.

To further verify the detection effect of UAV-YOLO in real scenarios, we shoot typical scenes such as road traffic in the city where we are located by utilizing the camera carried by the UAV, and detected the image data transmitted back from the UAV by using the detection method proposed in this paper, and some examples of the detection results are shown in Fig 13. From the final detection results, the UAV-YOLO is able to detect multiple types of targets in the image data well and maintains high real-time performance in both low-altitude and high-altitude scenarios. The above detection results also further demonstrate that UAV-YOLO can fully meet the requirements of target detection tasks in UAV aerial images.

## 5. Conclusions

This paper proposes a multi-scale UAV aerial image detection method UAV-YOLO based on adaptive feature fusion. Firstly, to address the problem of accurately and effectively extracting the feature information of small targets in UAV aerial images, an AFEM module is proposed to adaptively adjust the region of the receptive field of the convolution kernel according to the detected target, so as to obtain more feature information of small targets. In addition, this module can provide space and channel dimension attention to each target in the image. This can help to reduce the interference of background noise and to obtain more accurate and effective small target feature information. Secondly, to solve the problem of losing the shallow feature information of small targets in UAV aerial images, the SBiFPN network structure is designed to automatically adjust the weight of feature fusion between interrelated levels. This enables the full use of the shallow feature information such as texture and location which is conducive for the detection of small targets. Finally, to solve the problem of too many small targets in the aerial image as well as the scale that changes greatly in the aerial image during detection, four different detection scales are used to adapt to the detection of each target in the image. This helps to further improve the network’s ability to perceive small targets. The method proposed in this paper is tested by the VisDrone test set. The results show that compared with other existing UAV aerial image detection methods, UAV-YOLO achieves the best detection results in six categories such as pedestrians, truck, and cars, and achieves the best mAP value of 38.5%. The test results in a variety of scenarios show that the proposed method has good detection performance and can be better applied for the detection of UAV aerial image targets.

However, although our designed network has achieved more satisfactory detection results on the visdrone dataset, the detection accuracy of the existing detection methods for each target of UAV aerial images has been maintained at a low level, so next we will continue to build on this foundation to improve further the detection accuracy of the detection network for UAV aerial images.
